# Modified Atkins diet in adult with refractory epilepsy: A controlled randomized clinical trial

**Published:** 2017-04-04

**Authors:** Mohammad Zare, Ali Asghar Okhovat, Ahmad Esmaillzadeh, Jafar Mehvari, Mohammad Reza Najafi, Mohammad Saadatnia

**Affiliations:** 1 Isfahan Neurosciences Research Center, Alzahra Research Institute, Department of Neurology, Isfahan University of Medical Sciences, Isfahan, Iran; 2 Department of Neurology, Sina Hospital, Tehran University of Medical Sciences, Tehran, Iran; 3 Food Security Research Center, Department of Community Nutrition, School of Nutrition and Food Sciences, Isfahan University of Medical Sciences, Isfahan, Iran

**Keywords:** Epilepsy, Drug Refractory, Modified Atkins Diet, Adult

## Abstract

**Background:** The usefulness of the modified Atkins diet (mAD) in refractory epilepsy in adults has been rarely investigated. We aimed to evaluate the efficacy of mAD in adult with refractory epilepsy.

**Methods:** In a controlled randomized clinical trial, we enrolled 66 refractory adult epileptic cases from February 2010 to December 2012. The patients were randomly divided into two groups, case groups (22 patients) used antiepileptic drugs and mAD and control group (32 patients) only use antiepileptic drugs. The primary outcome was at least 50% decrement in seizure frequency after 2 months of therapy.

**Results:** No significant difference was shown in our data between groups regarding baseline characteristic. The differences of mean seizure attack after 2 months (P < 0.001). (17.6%) had > 50% seizure decrease at 1 and after 2 months and 12 (35.3%) had 50% decrease in seizure frequency. Furthermore, in mAD group, the mean urinary ketone positivity was 1.75 ± 0.28 and increasing liver enzyme was shown 5 cases (14.7%) in mAD group and 5 cases (15.6%) in control group (P < 0.050).

**Conclusion:** The mAD may be effective as a cotherapy treatment for adults with refractory epilepsy and decrease 2.19 times seizure frequency in comparison with control groups. Trials with the more tolerant dietary regime, with larger sample size and longer duration, should be performed in future.

## Introduction

Despite the appropriate consummation of several anticonvulsants, 10-30% of patients with epilepsy have refractory seizures. The ketogenic diet is a separately considered and severely controlled high-fat (80%), low protein (15%), and low carbohydrate (5%) diet used for the management of refractory seizures.^1^

The Atkins diet (AD) also limits carbohydrates, but unlike the ketogenic diet, it does not restrict usage of calories or proteins. The modified AD (mAD) provokes ketosis, but without neither fluid, calorie and protein limitation nor the requirement for fasting, food evaluating and hospitalization.^2^^,^^3^ In the past few years, there have been studies that Atkins and the mAD can be potentially applied as cotherapy for patients with refractory epilepsy.^2^^-^^7^

The use of dietary therapy treatment for epilepsy is technologically simple, and there are many studies about children that have showed the usefulness of mAD in refractory epilepsy.^4^^-^^6^ However, the mAD is rarely offered to adults.^7^ We aimed to assess the efficacy of mAD in adults with refractory epilepsy in a controlled randomized clinical trial.

## Materials and Methods

In a controlled randomized clinical trial, we compared the efficacy and tolerability of mAD in adults with refractory epilepsy. This study was registered in the Iranian Registry of Clinical Trials under ID number IRCT138803051949N1. We enrolled 66 refractory adult epileptic cases, aged from 18 to 57 years, who referred to the Adult Neurology Clinic of Kashani Hospital from February 2010 to December 2012. The inclusion criteria were the age of ≥ 18 years and refractory epilepsy (two or more seizures attacks every month in spite of treatment with at least two appropriate antiepileptic drugs). No changes were made in the study participants’ medications or treatment plans until informed consent was obtained. Exclusion criteria were previous use of the AD or mAD for > 1 week, previous use of the ketogenic diet within the past year, patients with kidney, heart, renal disease or hypercholesterolemia, patients with history of coronary heart disease, cerebrovascular disease, peripheral vascular disease, atherosclerosis, previous myocardial infarctions, or renal dysfunction, pregnant individuals, body mass index (BMI) below 18.5, status epilepticus within the past 6 months, 2 week seizure-free period within the past 6 months. This study was accepted by the Ethics Committee of Isfahan University of Medical Sciences, Iran. 

**Figure 1:**
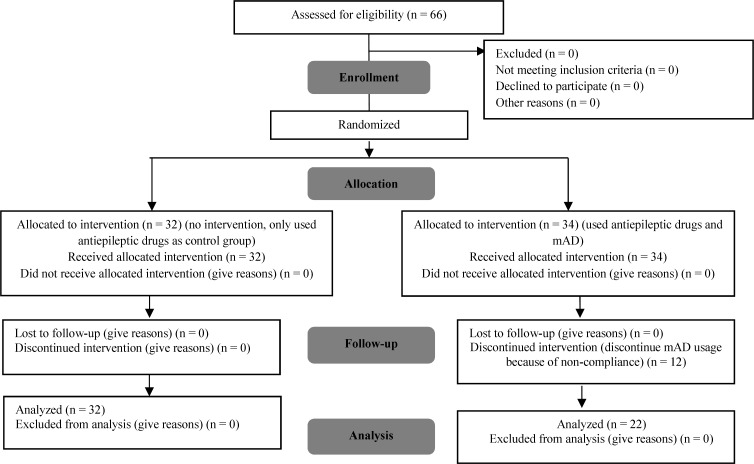
Flowchart consort

The patients were randomly separated into two groups according to random number table, case groups used antiepileptic drugs and mAD and control group only used antiepileptic drugs. After enrollment, case groups referred to nutritionist for education of mAD by using simple comprehensible terms and followed them for 2 months. The mAD was customized to the cultural and financial status of the families (Figure 1). 

mAD: Modified Atkins diet

The diet in case group was initiated with carbohydrates limited to 15 g/day, without any changes or limitations in calories and liquid in dietary pattern, it means 4-6% carbohydrate, 20-30% protein, and 60-70% fat. Before launching the diet, the patients were asked to record the frequency of their seizures (number/day) for 1 month. A monthly calendar with instructions to document seizures daily was provided. During 1 month every week by phone and after 1 month, calendars by clinic visit were reviewed. We measured the weight, height, lipid profile, serum electrolyte and liver function tests at onset and monthly during 2 months. Urinary ketones and weight were measured weekly for 2 months. However, the patients in case group were recommended to use high-fat food. Antiepileptic drug therapy continued unchanged for at least the 2 months, but when necessary, the drugs were changed in both groups. Low carbohydrate, multivitamins, and calcium supplementation were given to all patients. The least follow-up was 2 months and after that, if the diet was useful, the regimen was continued. The primary outcome was at least 50% decrease in seizure frequency after 2 months of therapy; the secondary outcome was the effects of diet on weight loss and ketone body. 

**Figure 2:**
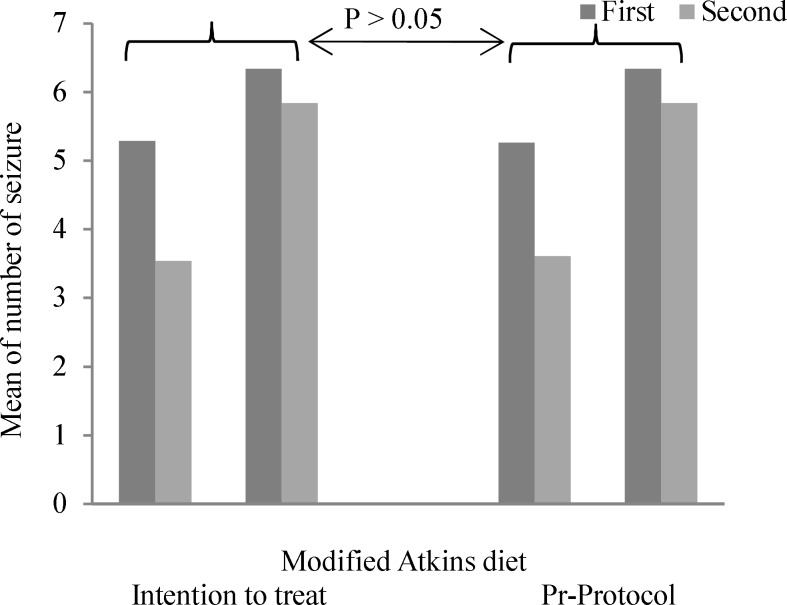
Comparison of change in mean number of seizure per month between intention to treat and pre-protocol analysis

Data on seizure frequency, age of the patients at the onset of epilepsy, classification of seizures, medications profile, demographic data, and results of serial biochemical assessment were collected and entered into SPSS software (version 18, SPSS Inc., Chicago, IL, USA). We employed an intent-to-treat (ITT) analysis for outcomes which were analyzed using independent sample t-test, paired sample t-test and chi-square except fisher test. Here, A two-tailed P < 0.050 was considered statistically significant. We compared the two groups concerning the study outcomes based on the per-protocol and ITT principles (Figure 2).

## Results

In this study, the control group includes 21 males (65.6%) and 11 females (34.4%) with the average age of 27.2 ± 7.3 years and mAD group composed of 34 males (70.6%) and 10 females (29.4%) with the average age of 29.4 ± 8.8 years, while both groups are the same in terms of age and sex without any significant differences statically (P > 0.050). Furthermore, between both groups there is no significant differences in terms of other factors such as duration of epilepsy, number of attack in month prior of study, family history of epilepsy, and so on (P > 0.050) (Table 1).

**Table 1:** Baseline and clinical characteristics of patients in two groups

**Variables**	**mAD group (n = 34)**	**Control group (n = 32)**	**P**
Age (year) (mean ± SD)	29.4 ± 8.8	27.2 ± 7.3	0.280
Sex [n (%)]			
Male	24 (70.6)	21 (65.6)	0.430
Female	10 (29.4)	11 (34.4)	
Duration of epilepsy (year) (mean ± SD)	17.80 ± 10.6	14.09 ± 7.50	0.100
Number of drugs (mean ± SD)	2.80 ± 0.98	3.03 ± 1.06	0.550
Number of attack in month prior of study (mean ± SD)	8.50 ± 7.00	6.50 ± 3.20	0.140
BMI prior of study (mean ± SD)	23.07 ± 3.60	22.95 ± 1.80	0.860
Past history of febrile convulsion [n (%)]	4 (11.8)	6 (18.8)	0.320
Family history of epilepsy [n (%)]	6 (17.6)	8 (25.0)	0.330
Type of seizure [n (%)]			
Complex partial	18 (52.9)	18 (56.3)	0.490
Generalized tonic clonic	16 (47.1)	14 (43.7)	

BMI: Body mass index; mAD: Modified Atkins diet; SD: Standard deviation

**Table 2:** Clinical characteristics of patients in which use of intention to treat was reported in two groups

**Variables**	**Month**	**mAD group ** **(n = 34)**	**Control group ** **(n = 32)**	**P** *
Number of seizure attacks per months (mean ± SD)	First	5.26 ± 2.81	6.34 ± 3.06	0.141
	Second	3.61 ± 1.88	5.84 ± 2.92	< 0.001
P**		0.006	0.001	
50% reduction in seizure frequency [n (%)]	First	6 (17.60)	0 (0)	0.037
	Second	12 (35.30)	0 (0)	0.001
P**		0.084	-	
Seizure free [n (%)]	First	0 (0)	0 (0)	-
	Second	0 (0)	0 (0)	-
P**		-	-	
BMI (kg/m^2^) (mean ± SD)	First	23.07 ± 3.60	22.95 ± 1.80	0.860
	Second	22.32 ± 3.52	23.02 ± 1.94	0.365
P**		0.038	0.237	
Increasing cholesterol level during 2 months [n (%)]		7 (20.59)	0 (0)	0.004
Increasing liver enzyme during 2 months [n (%)]		5 (14.71)	5 (15.62)	0.007
The mean urinary ketone positivity during 2 months (mean ± SD)		1.75 ± 0.28	-	-

SD: Standard deviation; mAD: Modified Atkins diet

* Significant level for comparing two groups in each months,

** Significant level for comparing two month in each groups.

Of participants, who started the mAD, due to discontinuing mAD usage or non-participation to final follow-up, 12 of them were excluded and 22 (64.7%) participants continued in the study. 6 (17.6%) had > 50% seizure reduction at 1 month using ITT analysis. After 2 months, 12 (35.3%) had > 50% reduction in seizure frequency using ITT analysis, and anybody was seizure-free. Furthermore, the mean of patients’ BMI in the 1^st^ month has no significant differences (P = 0.860) and in mAD group; in the 2^nd^ month, BMI had a significant reduction from 23.07 ± 3.60 to 22.32 ± 3.52 kg/m^2^ (P = 0.038). While in the control group, the mean of BMI had an increase from 22.95 ± 1.80 to 23.02 ± 1.94 kg/m^2^ but this increase was not significant statistically (P = 0.237). Furthermore, in mAD group within 2 months increasing cholesterol level has been seen in seven cases (20.0%) compared to control group suggests no increase in cholesterol level. Moreover, in mAD group, increasing liver enzyme has been seen in five cases (14.71%) compared to five cases (15.62%) in control group that shows a significant deference between two groups (P < 0.050). The mean urinary ketone positivity was reported only in mAD group 1.75 ± 0.28 (Table 2).

Finally, as figure 2 shows, both groups suggest no significant differences in terms of change in mean number of seizure per month and by taking into account two methods of intention to treat and pre-protocol analysis (P > 0.050).

## Discussion

This open label, prospective randomized clinical trial has revealed that the mAD appears to be an effective and well-tolerated management for adults with refractory seizures. At the end of the 2^nd^ month, 45.5% of patients had > 50% seizure decrease. It appears that mAD co-therapy can decrease 2.19 times more seizure reduction in comparison with control groups. 

The results of this small, open label, prospective and randomized clinical trial study agree with previous findings,^7^^-^^9^ which suggest that some benefits exist for the use of the mAD as a form of cotherapy in the managing of intractable epilepsy in an adult population. Therefore, data on mAD treatment in adults are limited. Only three open-label reports have shown the use of the mAD exclusively for adults.^7^^-^^9^ A literature review of studies which include data specifically on individuals aged > 18 on the mAD is shown in table 3. Data from three studies showed, on average, 9 of 32 (28.1%) adults achieved > 50% seizure reduction; among these 32 none of them became seizure-free. No specific data were provided for the adults included in some studies; but for those with seizure reduction, the mean time to improvement was 2 weeks (range: 1-8 weeks).^7^ However, to our literatures, this is the first randomized clinical trial, showed that mAD cotherapy can decrease 2.19 times more seizure reduction in comparison with control groups for adults with refractory seizures. 

**Table 3:** Summary of studies involving adults treated with the modified Atkins diet (mAD) in refractory seizure patients

**References**	**Study type**	**Number**	**Ages ** **(year)**	**Diet type**	**> 50% decrease (%)**	**Seizure ** **free (%)**	**End point ** **(month)**	**% dropout before ** **end of study**	**Adverse side effects**
Kossoff, et al.^7^	Prospective	30	18-53	mAD (carbohydrate restriction 15/day)	47% had a *> *50% seizure reduction after 1 and 3 months on the diet; 33% after 6 months.	0	6 months	53	Lethargy, weight loss, elevated total cholesterol, leg swelling
Carrette, et al.^8^	Prospective	8	31-55	mAD (carbohydrate restriction 20/day)	33% after 6 months	0	6 months	62.5	Vomiting, headache, nausea, diarrhea, constipation, weakness, weight loss, elevated total and LDL cholesterol
Smith, et al.^9^	Prospective	18	18-55	mAD (carbohydrate restriction 20/day)	12% had a > 50% seizure reduction after 3 months; 28% after 6 months, and 21% after 12 months	0	12 months	22	Weight loss (desired), left arm jerks

mAD: Modified Atkins diet

A positive correlation was observed between the mean urinary ketone level and > 50% seizure reduction in case group. However, the previous study showed that ketone levels had no correlation with improved efficacy in adults.^7^^,^^9^

In this study, may be due to high consumption of lipid and fatty liver consequently, cholesterol and liver enzyme level were higher in case group. Gastrointestinal complaints and unfavorable lipid profiles (low density lipoprotein and total cholesterol increment) were side effects of mAD in another study.^7^^,^^10^^,^^11^ We showed no significant weight loss in mAD group, however, weight loss was more common in adults who initially requested to lose weight.^7^ One study showed a correlation of BMI reduction and diet effectiveness,^7^ but another study did not find this.^9^

Nevertheless, it appears that tolerability of the mAD in adults is similar to that of the ketogenic diet and long-term side effects of the mAD are unknown in adults and should be evaluated in future study with larger sample size and longer duration.

In this study, we showed 35.2% of patients discontinued the mAD treatment. Data from other three studies showed that, on average, 46.6% of patients discontinued the treatment.^7^^-^^9^ The main cause for treatment discontinuation appears to be the lack of efficiency.^7^^-^^9^ The percentage dropout with the ketogenic diet ranged from 10% to 88% (mean = 53%),^7^^-^^9^ despite limited sample sizes; retention appeared higher on the mAD than on the ketogenic diet, and so more tolerant regimens may be proposed as feasible alternatives for older people. Nevertheless, more than 50% of patients in various studies were motivated to maintain it as long as seizures were reduced.^7^^-^^9^

However, this study has some limitation, first of all the small sample size, the second the two groups were not similar according to antiepileptic drug therapy.

## Conclusion

The mAD may be effective as a cotherapy treatment for almost the half of adults with refractory epilepsy that decrease 2.19 times seizure frequency in comparison with control groups. The retention levels are poor (near to 40%). However, studies are limited about adults and trials with more tolerant dietary regime, with larger sample size and longer duration should be performed in the future.

## Notes:

**How to cite this article:** Zare M, Okhovat AA, Esmaillzadeh A, Mehvari J, Najafi MR, Saadatnia M. Modified Atkins diet in adult with refractory epilepsy: a controlled randomized clinical trial. Iran J Neurol 2017; 16(2): 72-7.
